# Relevance of the Exocyst in Arabidopsis *exo70e2* Mutant for Cellular Homeostasis under Stress

**DOI:** 10.3390/ijms24010424

**Published:** 2022-12-27

**Authors:** João Neves, João Monteiro, Bruno Sousa, Cristiano Soares, Susana Pereira, Fernanda Fidalgo, José Pissarra, Cláudia Pereira

**Affiliations:** 1 Faculdade de Ciências, Universidade do Porto, Rua do Campo Alegre s/n, 4169-007 Porto, Portugal; 2 GreenUPorto-Sustainable Agrifood Production Research Centre and Inov4Agro—Institute for Innovation, Training and Sustainability of Agrifood Production, Rua do Campo Alegre s/n, 4169-007 Porto, Portugal

**Keywords:** abiotic stress, exocyst, EXPO, Antioxidant system, endomembrane system, protein trafficking

## Abstract

Plants must adapt to cope with adverse environmental conditions that affect their growth and development. To overcome these constraints, they can alter their developmental patterns by modulating cellular processes and activating stress-responsive signals. Alongside the activation of the antioxidant (AOX) system, a high number of genes are expressed, and proteins must be distributed to the correct locations within the cell. The endomembrane system and associated vesicles thus play an important role. Several pathways have been associated with adverse environmental conditions, which is the case for the exocyst-positive organelle—EXPO. The present work, using Arabidopsis mutants with T-DNA insertions in the gene *EXO70*, essential for EXPO vesicles formation, was designed to characterise the anatomical (morphology and root length), biochemical (quantification of stress markers and antioxidant system components), and molecular responses (gene expression) to abiotic stresses (saline, drought, oxidative, and metal-induced toxicity). The results obtained showed that mutant plants behave differently from the wild type (WT) plants. Therefore, in the *exo70* mutant, morphological changes were more noticeable in plants under stress, and the non-enzymatic component of the antioxidant system was activated, with no alterations to the enzymatic component. Furthermore, other defence strategies, such as autophagy, did not show important changes. These results confirmed the EXPO as an important structure for tolerance/adaptation to stress.

## 1. Introduction

Climate change is a major threat to crop development, as it affects multiple aspects of agricultural systems, such as water availability, soil fertility, pathogen spread, and host susceptibility. The combination of these factors can cause crop failures, leading to food insecurity, in times when the demand for agricultural products continues to rise [1,2]. In fact, due to their sessile nature, plants are in a constantly changing environment, which is often unfavourable to their development [3]. As one of the most susceptible organisms to abiotic stresses [2,3], plants have evolved to be capable of adapting and taking advantage of these situations through multiple signalling pathways that lead to physiological adjustments, such as protein accumulation, alteration of developmental patterns, and antioxidant (AOX) system activation [3,4,5]. Upon exposure to stress, multiple metabolic changes occur. One of the most common is the overaccumulation of reactive oxygen species (ROSs), the generation of which is enhanced in response to different biotic and abiotic stresses [5,6]. ROSs play a dual role depending on their levels in plant cells: at low concentrations, they serve as important intracellular signals, while at higher concentrations, they become phytotoxic and capable of interacting with all kinds of organic molecules, jeopardising plant cell homeostasis. To prevent an unregulated increase in these compounds, plants have developed an efficient AOX system to keep cells’ redox states under control. When there is a disproportion between production and elimination of ROSs, an oxidative stress situation takes place, leading to cellular and subcellular damage [7,8]. To ensure redox homeostasis, the AOX system must act effectively, through the joint action of both non-enzymatic and enzymatic mechanisms. The non-enzymatic component comprises diverse molecules and low mass metabolites, such as ascorbic acid (AsA), glutathione (GSH), proline (Pro), and phenolic compounds [5]. These molecules can directly neutralise, transform, or remove ROSs, allowing the cell to sense and maintain the redox homeostasis. However, the efficiency of these AOX players depends on a conjuncture of factors, such as the type of stress, length of exposure, intensity, plant species, genotype, affected organ, among other physiological traits [7]. On their turn, and working together with the AOX metabolites, the enzymatic components provide a complex and multifaceted protection mechanism to maintain cell homeostasis by avoiding oxidative-induced damage and supporting plant development [5]. This enzymatic part includes three major enzymes: superoxide dismutase (SOD; EC 1.15.1.1), catalase (CAT; EC 1.11.1.6), and ascorbate peroxidase (APX; EC. 1.11.1.11). Overall, the interdependence between non-enzymatic and enzymatic AOX players provides plants with a sophisticated defence network, allowing them to properly balance ROS production and avoid cellular damage. However, as stated previously, given the magnitude (e.g., intensity and frequency) of the stress exposure, the AOX system is not always capable of ensuring redox homeostasis [5].

Alongside the AOX system activation, other cell processes are induced or re-calibrated in order to cope with adverse situations. Thus, upon stress-sensing mechanisms, the endomembrane system is also modulated, including its natural morphology and protein effectors. The endoplasmic reticulum (ER) is the first organelle to be affected, by activating the unfolded protein responses (UPRs) and ER-derived autophagy [9,10,11]. Then, the Golgi complex generates a large number of vesicles under stressful situations, presenting a hypertrophic state [12]. The lytic vacuole (LV), on its turn, displays an adaptive response dependent on the type of stress. For example, in cases of Mg^2+^ toxicity, certain kinases are activated to detoxify the excessive amount of ions [13]. In addition, in the presence of ROS, the LV stores GSH as a mechanism to protect the cell from a shift in cytosolic GSH redox potential [14]. The protein sorting vacuole (PSV) has an increase in cysteine proteases, which normally are responsible for the degradation of storage proteins during plant development. However, studies have demonstrated that they might be triggered by stress, providing a new perspective on the cysteine proteases in stress response [15,16]. Moreover, genes that encode proteins involved in the PSV route are enhanced, while the LV route is less chosen, showing a preference for a more reserved state [12]. Considered by several authors as part of the endomembrane system, exocytosis is one of the fundamental processes for cell survival, being involved in the regulation of several key factors in cell development, such as cell polarity/morphogenesis, protein recycling, cell wall biogenesis, and interactions during adverse environmental conditions. Therefore, it is characterised as an important regulatory hub of the endomembrane dynamics [17]. The exocyst-positive organelle (EXPO) is a double-membrane organelle responsible for the transport of cargos to the plasma membrane. The EXPO is part of the unconventional protein secretion (UPS) routes, since the model proposed for its action requires the sequestration of cytosolic leaderless secretory proteins (LSP) that will be then fused with the plasma membrane (PM) [18,19,20,21]. The EXPO is a complex formed by proteins of the EXO70 family, and is responsible for exocyst formation. In land plants, this family is encoded by a large number of *EXO70* paralogues (23 in Arabidopsis), while in other eukaryotic organisms, there is only one [22,23]. This trait can be explained by the different needs that plant cells present compared with other eukaryotic cells, such as cell-specific exocytosis within differentiating plant tissues and exocytosis related to biotic and abiotic stress responses [17,21,22,24].The first exocyst subunit described in Arabidopsis was *AtExo70E2*. This marker is fundamental for the assembly of the exocyst, since it is the one that recruits the other subunits. However, the mechanism behind this process is still undefined [21]. In a recent study, we demonstrated that, under abiotic stress conditions, the expression of the gene that encodes AtEXO70E2 is highly upregulated [12]. This represents a hint that under adverse environmental conditions, EXPO-mediated exocytosis is enhanced, showing that UPS responses are more pronounced and may play a role in the plant’s defence system.

Thus, this work was carried out as a follow-up study, which seeks to explore how abiotic stress affects protein sorting mechanisms, namely the EXPO-mediated sorting, aiming to provide new insights into how proteins involved in protein sorting are relevant to the stress response. To accomplish this, an *exo70e2* T-DNA *Arabidopsis thaliana* line was characterised in combination with different abiotic stresses. These plants were analysed according to different perspectives to obtain an integrated vision of these processes: biometric parameters, expression analysis of endomembrane effectors from the related transport routes, and biochemical characterisation of the AOX status. The data acquired from these experiments allowed us to gain a better understanding of the relevant role of EXPO in the stress response.

## 2. Results

### 2.1. exo70e2 T-DNA Plants Exhibit Developmental Defects under Stress

**Figure 1:**
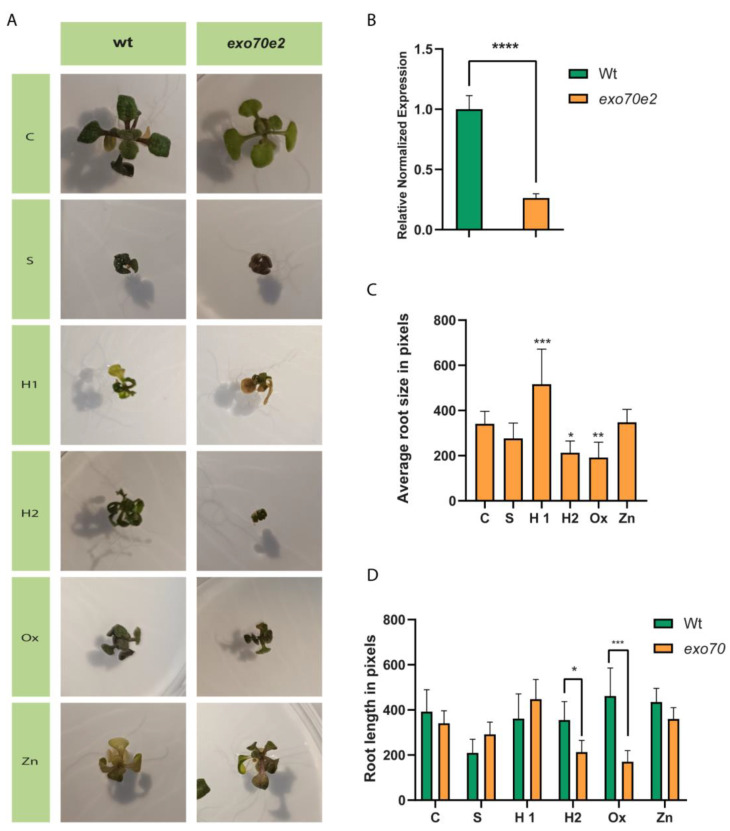
Biometric analysis of *exo70e2* T-DNA Arabidopsis line and comparison with wild type (wt). (**A**) Comparison of *Arabidopsis thaliana* seedlings from wt and *exo70e2* germinated under stress conditions. (**B**) Bar plot showing the relative normalised expression, by qPCR analysis, of *AtEXO70e2* in wt plants and in the *exo70e2* background. (**C**) Bar plot showing the average root size of *exo70e2* under stress conditions, compared to control. (**D**) Bar plot of the average root size of *exo70e2* plants, germinated under stress, compared to wt plants in the same conditions. All data presented were acquired 12–14 days post-germination, with three or more biological replicates (n ≥ 3). As shown in (**B**), the exo70e2 line is a knockdown for this gene; however, it significantly impaired the development of the seedling, particularly when exposed to high-dose drought stress and oxidative conditions. Statistically experimental values are represented by: * (*p* value ≤ 0.05), ** (*p* value ≤ 0.01); *** (*p* value ≤ 0.001); **** (*p* value < 0.0001). C—control; S—salt stress (50 mM NaCl); H1—drought stress (50 mM mannitol); H2—drought stress (10 mM mannitol); Ox—oxidative stress (0.5 mM hydrogen peroxide); Zn—metal poisoning stress (150 µM zinc sulphate).

Seeds from *exo70e2* mutant plants were germinated, alongside wild type (wt) plants, under different abiotic stress conditions: salinity (S), drought (H1 and H2), oxidative (Ox), and Zn toxicity (Zn). In the *exo70e2* T-DNA line, morphological changes were found when compared to the wt. In control seedlings, the presence of purple stains visible in leaves indicated areas accumulating anthocyanins, and the mutant showed some developmental delay (Figure 1A C). Plants with this genotype grown under S, H1, and H2 treatments had similar morphological responses, such as signs of developmental delay (Figure 1A S, H1, and H2), particularly in the H2 situation. A different response was observed when seedlings were under oxidative stress, where a developmental delay was registered, since in the wt, this stress situation did not show major developmental alterations (Figure 1A Ox). Moreover, Zn-exposed seedlings presented evident symptoms of leaf chlorosis compared to the control (Figure 1A Zn). The analysis of primary root length showed a significant increase in H1 (51%) and a significant decrease in H2 (37%) and Ox (38%) when compared with the control condition (Figure 1C). However, in direct comparisons of the root length of the mutant with the wt, significant differences were not observed in the control situation, but a decrease was found in H2 (40%) and Ox (67%) (Figure 1D).

### 2.2. Expression of EXPO-Related Genes Is Altered in the Mutant Background

**Figure 2:**
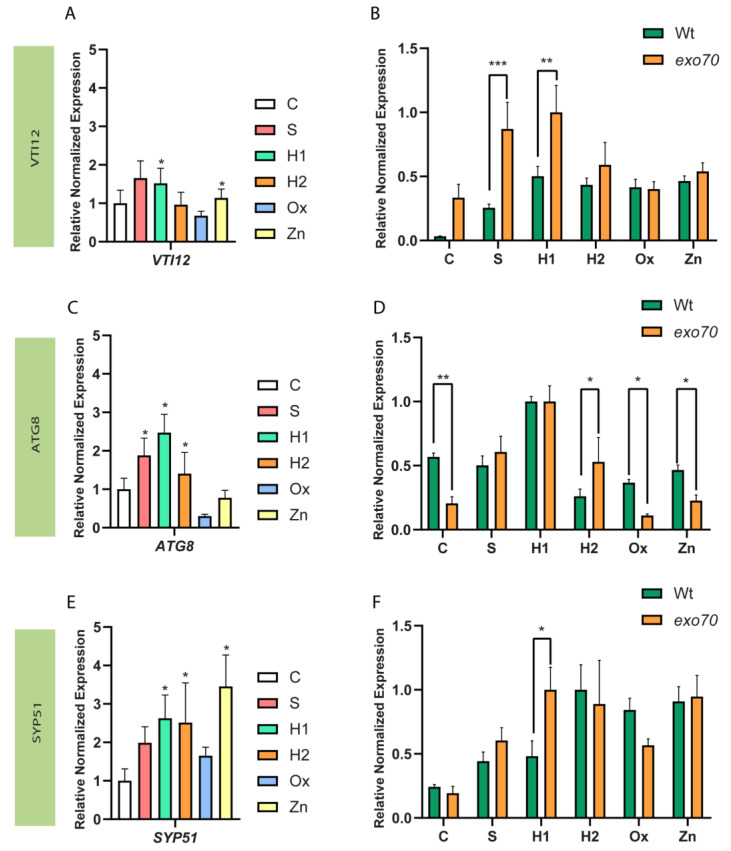
Expression analysis by qRT-PCR of EXPO-related genes in *exo70e2* plants in control and under stress. Bar chart analysis of relative normalised expression of *AtATG8* (**A**,**B**), *AtVTI12* (**C**,**D**), and *AtSYP51* (**E**,**F**), in the mutant background and under stress conditions. Analysis was performed by comparing each stress condition with the control (**A**,**C**,**E**) and by comparing each stress condition in wt and *exo70e2* plants (**B**,**D**,**F**). Expression of these genes varied with the stress condition in the mutant background, and significant changes were observed when the expression was compared with that of wt, in the same stress conditions. The outcome results from the evaluation of at least three experimental replicates (n ≥ 3) are presented. * Indicates statistically experimental values (*p* < 0.05), ** (*p* value ≤ 0.01), *** (*p* value ≤ 0.001), calculated using one-way ANOVA statistical tests. C—control; S—salt stress (50 mM NaCl); H1—drought stress (50 mM mannitol); H2—drought stress (10 mM mannitol); Ox—oxidative stress (0.5 mM hydrogen peroxide); Zn—metal poisoning stress (150 µM zinc sulphate).

Initially, a qPCR analysis was performed to evaluate whether this T-DNA line corresponded to a knockdown or a knockout line, as the insertion was located in an intron. The results showed that the line used in this study was a knockdown, with a significant decrease in the expression of *EXO70E2* compared to the wt (Figure 1B). Since EXO70E2 is involved in the exocyst assembly, and this structure is frequently associated with stress and defence mechanisms, the expression of genes related to this route or with similar roles was evaluated. The genes selected for analysis were *AtATG8* (autophagy marker), *AtVTI12* (responsible for exocyst docking at the vacuole), and *AtSYP51* (important for protein transport to the vacuole and often associated with stress responses), and their relative expression was compared to the one obtained for the wt plants, both under control conditions and under stress (Figure 2).

*AtVTI12* expression, under stress and in the mutant background, showed a significant overexpression in H1 and Zn situations compared to control conditions (Figure 2A). No significative changes were observed for the other stress treatments (Figure 2A). When comparing the expression levels between the mutant and wt plants, it was possible to observe that, even under control conditions, the expression of *AtVTI12* was increased in the *exo70e2* plants (Figure 2B). This increase can also be observed in S and H1 situations, without major changes in the other stress conditions (Figure 2B). Upon observing *AtATG8* expression under stress, and comparing it to that in the control in the mutant background, this gene was upregulated in S, H1, and H2 (Figure 2C). In fact, when comparing its expression in the *exo70e2* with that in wt plants, significant overexpression of *AtATG8* was observed in H2 (Figure 2D). Importantly, the expression of this gene in the control condition, along with in Ox and Zn conditions, was downregulated when compared to wt plants (Figure 2D).

### 2.3. Analysis of Biochemical Endpoints

**Figure 3:**
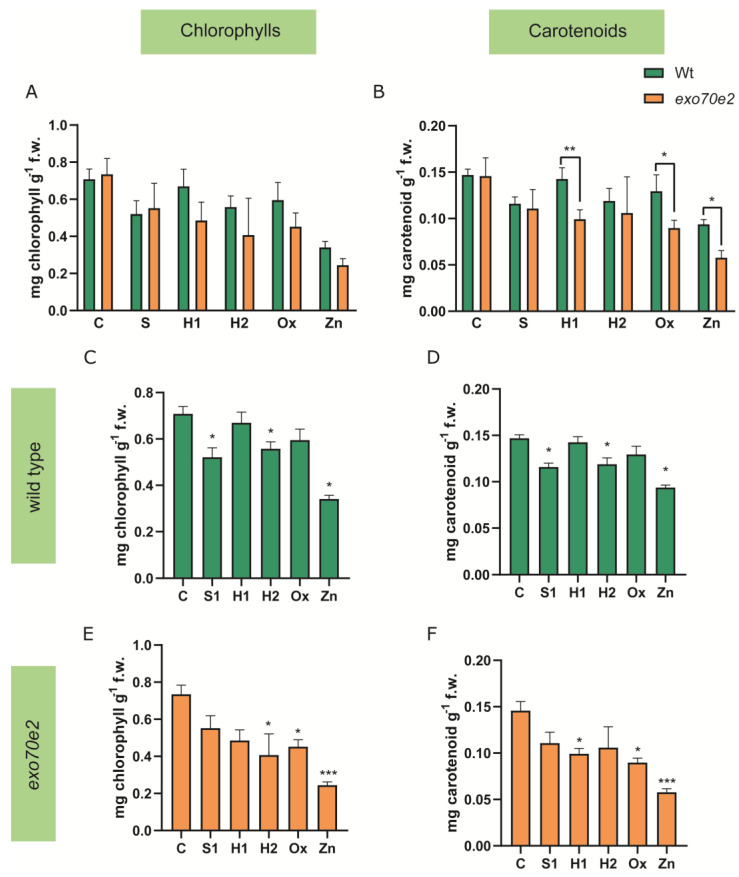
Total chlorophylls and carotenoids in *exo70e2* and wt plants, in control conditions and under stress. (**A**,**B**) comparison between the wt and *exo70e2* plants. (**C**,**E**) Chlorophyll content in wt and *exo70e2* plants, respectively. (**D**,**F**) Quantification of carotenoids content from wt and *exo70e2* plants, respectively. Results are presented as mean ± standard deviation (SD) and the outcome from the evaluation of at least three experimental replicates (n ≥ 3). A significant decrease in the amount of pigments can be detected in the Zn stress group, with the exo70e2 background. * (*p* value ≤ 0.05); ** (*p* value ≤ 0.01); *** (*p* value ≤ 0.001); according to the one-way ANOVA statistical tests. C—control; S—salt stress (50 mM NaCl); H1—drought stress (50 mM mannitol); H2—drought stress (10 mM mannitol); Ox—oxidative stress (0.5 mM hydrogen peroxide); Zn—metal poisoning stress (150 µM zinc sulphate).

The photosynthetic pigments (total chlorophylls and carotenoids) were quantified in wt and *exo70e2* mutant leaves, and both showed similar responses. Comparing wt with mutant plants, no changes were observed for the chlorophyll content in all conditions tested (Figure 3A), while the amount of carotenoids decreased slightly in the Exo70e2 background when exposed to H1, Ox, and Zn (Figure 3B). Under stress conditions in wt plants, chlorophylls and carotenoids exhibited a significant decrease in S1 (26% and 17%), H2 (20% on both), and Zn (51% and 40%) conditions (Figure 3C,D). *Exo70e2* plants demonstrated a decrease in the amount of both pigments under Ox (38% and 36%) and Zn (67% and 59%) conditions. Plants under H1 stress only showed a significant decrease in carotenoids (29%), and plants under H2 conditions showed a decrease in chlorophylls (45%) (Figure 3E,F).

**Figure 4:**
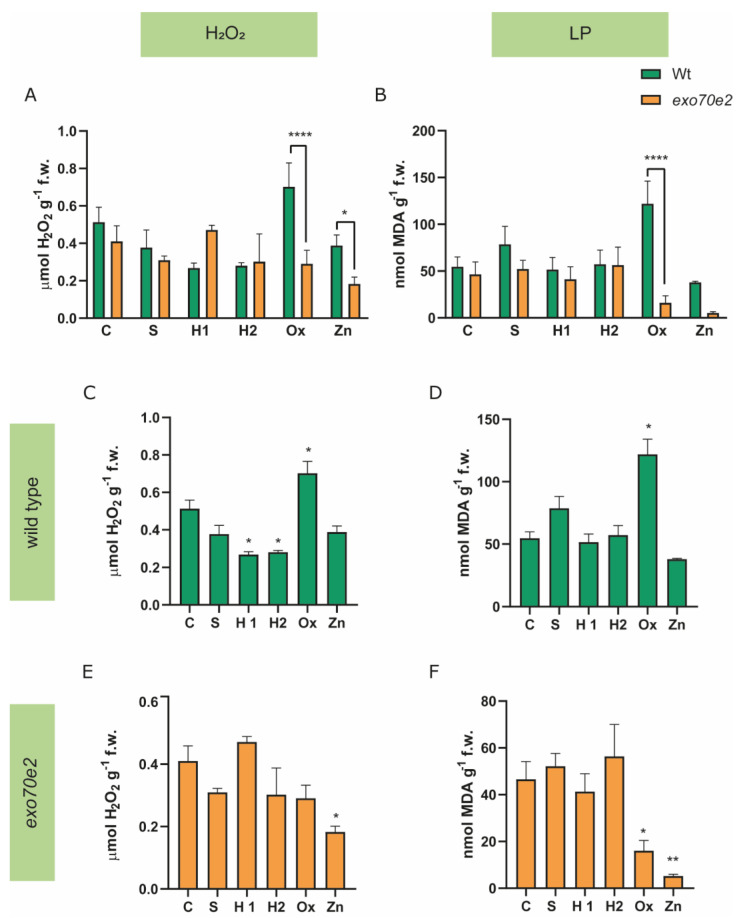
**H_2_O_2_ and LP activity (expressed as nmol MDA g^−1^ f.w.) in *exo70e2* and wt plants in control conditions and under stress.** (**A**,**B**) Comparison between the wt and *exo70e2* plants. (**C**,**E**) H_2_O_2_ quantification from wt and *exo70e2* plants under stress conditions, respectively. (**D**,**F**) LP activity calculated for wt and *exo70e2* plants germinated under stress. Results are presented as mean ± standard deviation (SD) and results from the evaluation of at least three experimental replicates (n ≥ 3). No changes were observed for the mutant compared to wt under control conditions. However, a significant decrease in H_2_O_2_ and LP levels was observed for Zn stress in *exo70e2* plants, while in wt, no change was observed. The decrease in LP levels in Ox treatment in mutant plants, contrary to the increase observed in wt plants, is also noticeable. * (*p* value ≤ 0.05), ** (*p* value ≤ 0.01); **** (*p* value < 0.0001), according to the one-way ANOVA statistical tests. C—control; S—salt stress (50 mM NaCl); H1—drought stress (50 mM mannitol); H2—drought stress (10 mM mannitol); Ox—oxidative stress (0.5 mM hydrogen peroxide); Zn—metal poisoning stress (150 µM zinc sulphate).

To evaluate whether the treatment was affecting cells’ redox status and causing the occurrence of oxidative stress, hydrogen peroxide (H_2_O_2_, a ROS) and lipid peroxidation (LP) were quantified. Wt plants showed a decrease in the amount of H_2_O_2_ across treatments, though statistical relevance was only achieved for H1- (50%) and H2- (45%) treated plants. However, in Ox-treated plants, there was a significant increase (36%) in the amount of H_2_O_2_ (Figure 4C).

In *exo70e2* plants, although a general tendency for decreased values of H_2_O_2_ was found in all treatments compared with the controls, a significant reduction of about 56% was recorded only upon exposure to Zn (Figure 4E). The H_2_O_2_ contents for the two genotypes (wt, *exo70e2*) under control conditions were compared, but no significant changes were found (Figure 4A). Concerning LP levels, and comparing control plants, there were no significant differences between the wt and the T-DNA lines (Figure 4B). Regarding the behaviour of the wt plants under stress treatments, only the oxidative stress (Ox) induced a significant increase in MDA (123%). H1 and H2 treatments did not change this parameter; however, MDA levels showed a tendency to increase in S-treated plants and to decrease in Zn-treated plants, in relation to the control plants (Figure 4D). In *exo70e2* plants, there were no significant changes between the MDA amounts in S, H1, or H2 treated plants; however, under Ox conditions, there was a significant reduction in MDA amounts (65%), and an even higher decrease (88%) was observed in the Zn-treated plants when compared with the controls (Figure 4F).

### 2.4. Evaluation of the Antioxidant System in exo70e2 Mutant Plants

**Figure 5:**
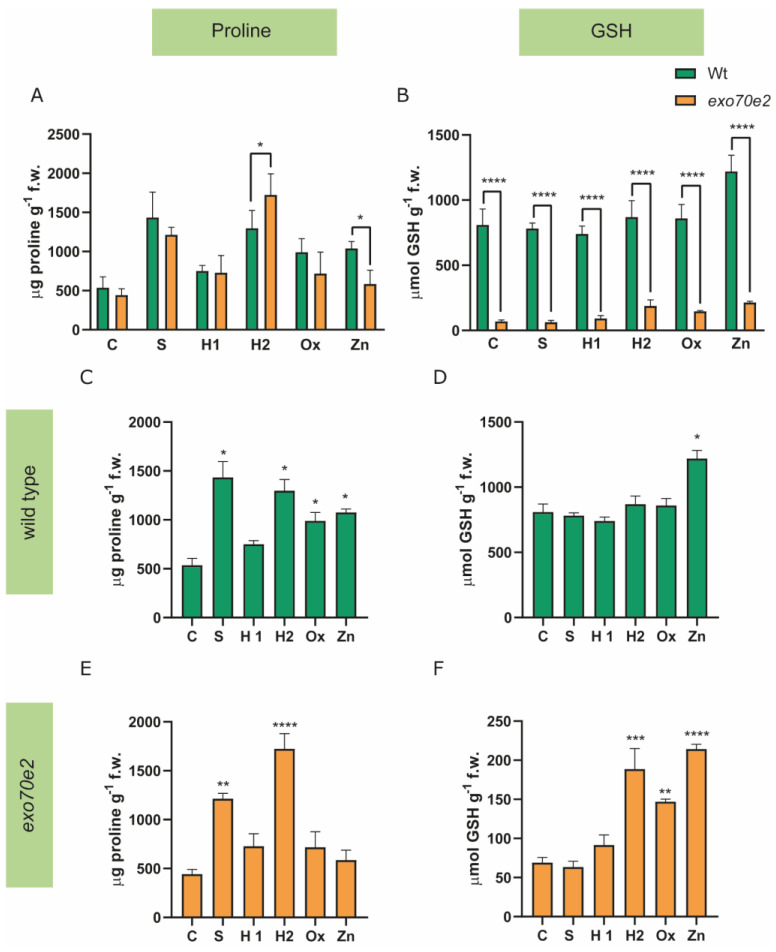
Proline (Pro) and GSH in exo70e2 plants in control and under stress. (**A**,**B**) Comparisons of Pro and GSH values between wt and exo70e2 plants. (**C**,**E**) Quantification of Pro accumulation in wt and exo70e2 plants, respectively, grown under stress. (**D**,**F**) GSH quantification in wt and exo70e2 plants, respectively, under stress. Results are presented as mean ± standard deviation (SD) of at least three experimental replicates (n ≥ 3). A significant decrease in GSH levels can be observed in exo70e2 plants in control conditions when compared to wt levels. An increase in GSH levels in H2, Ox, and Zn was also clear, along with an increase in Pro accumulation in S and H2 conditions. * (*p* value ≤ 0.05); ** (*p* value ≤ 0.01); *** (*p* value ≤ 0.001); **** (*p* value < 0.0001), according to the one-way ANOVA statistical tests. C—control; S—salt stress (50 mM NaCl); H1—drought stress (50 mM mannitol); H2—drought stress (10 mM mannitol); Ox—Oxidative stress (0.5 mM hydrogen peroxide); Zn—metal poisoning stress (150 µM zinc sulphate).

The non-enzymatic component was evaluated by quantifying the intracellular levels of Pro and GSH (Figure 5).

**Figure 6:**
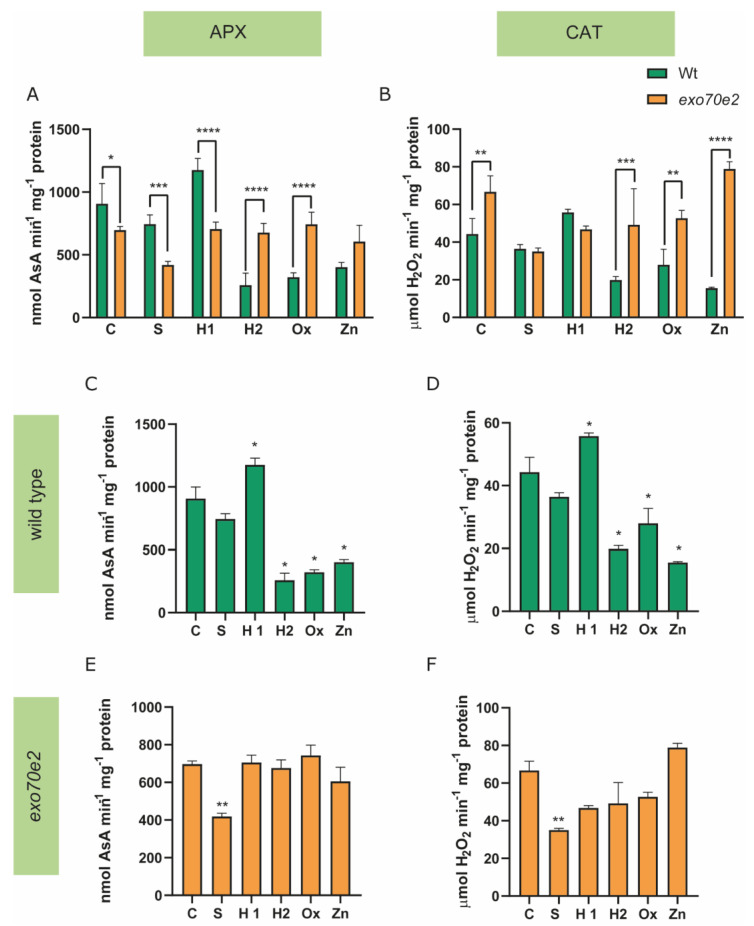
APX and CAT activity in exo70e2 plants in control and under stress. (**A**,**B**) Comparison of APX and CAT activity, respectively, in wt and exo70e2 plants. (**C**,**E**) APX activity calculated for wt and exo70e2 plants grown under stress. (**D**,**F**) CAT activity calculated for wt and exo70e2 plants grown under stress. Results are presented as mean ± standard deviation (SD) from the evaluation of at least three experimental replicates (n ≥ 3). No alterations in CAT and APX activity in control conditions between wt and exo70e2 plants were observed. APX and CAT activity was significantly reduced in wt plants under H2, Ox, and Zn conditions, while for the mutant background, no changes are observed. * (*p* value ≤ 0.05); ** (*p* value ≤ 0.01); ***(*p* value ≤ 0.001); **** (*p* value < 0.0001), according to the one-way ANOVA statistical tests. C—control; S—salt stress (50 mM NaCl); H1—drought stress (50 mM mannitol); H2—drought stress (10 mM mannitol); Ox—oxidative stress (0.5 mM hydrogen peroxide); Zn—metal poisoning stress (150 µM zinc sulphate).

Wt plants, in stressful situations, showed an increased accumulation of Pro compared to the controls (C), excluding the H1 treatment group, where no alteration was identified (Figure 5A). For instance, S- and H2-treated plants showed a 1.5-fold increase in Pro levels, while those exposed to Ox and Zn showed increases of approximately 0.8 and 1-fold, respectively (Figure 5C). In *exo70e2* plants, a significant increase was detected in the Pro amount compared with the controls, since in S- and H2-treated plants, the content of this amino acid significantly increased by 1.73-fold and 3-fold, respectively (Figure 5E). Finally, regarding GSH accumulation on control plants (wt and *exo70e2*), a significant decrease (91%) was observed in *exo70e2* plants (Figure 5B). Interestingly, wt plants under stress conditions only showed a significant change of GSH upon Zn exposure, with an increase of 50% recorded compared to the control condition (Figure 5D). Regarding *exo70e2*, alterations in GSH levels were observed in response to H2, Ox, and Zn conditions; additionally, the GSH amount increased significantly, by 170%, 112%, and 210%, respectively (Figure 5F). Alongside the quantification of the non-enzymatic component of the AOX system, the behaviour of the enzymatic one was also evaluated, by quantifying the activity of APX and CAT. No significant changes in APX levels were found when comparing control plants with *exo70e2* ones in relation to wt (Figure 6A). Wt seedlings showed a significant increase in APX activity in H1 (29%) and a significant decrease in H2 (71%), Ox (64%), and Zn (55%) treatments (Figure 6C). Concerning *exo70e2* plants, no significant alterations were observed, excluding S-treated plants, which showed a reduced APX activity of 40% compared to the control (Figure 6E). Regarding CAT levels in control plants, no significant changes were found between the wt and the mutant plants (Figure 6B). In wt plants under stress, CAT activity showed a significant increase in H1 (26%) and a decrease in H2 (55%), Ox (37%), and Zn (65%) treatments (Figure 6D). Concerning *exo70e2* plants, a significant reduction in CAT activity was found with S1 stress (47%), but no major changes were detected with the other treatments (Figure 6F).

## 3. Discussion

Nowadays, agriculture is frequently confronted with new and stronger challenges triggered by climatic alterations and human activities. These can substantially impact the agroecosystems, affecting the natural development of crops [1,2]. Therefore, plants must adapt to and take advantage of stressful situations in order to thrive by promoting metabolic, molecular, and cellular adjustments, such as protein trafficking, accumulation of proteins, and the activation of powerful AOX networks [3,5,16]. A former study from our laboratory showed that *Arabidopsis thaliana* wt seedlings exposed to abiotic stress conditions exhibited developmental delays and chlorosis in the leaves, especially under salt and metal-induced stress, respectively [12]. Here, we used T-DNA Arabidopsis plants’ knockdown of the *EXO70E2* gene and followed a similar stress-inducing scheme. Exo70E2 was shown to be crucial for the formation of the exocyst in plants [18]; thus, it is to expected that this route would be impaired in this mutant.

### 3.1. Characterisation of exo70e2 Mutant Line under Abiotic Stress

A thorough characterisation of *exo70e2* mutant plants was performed under stress, involving not only gene expression, but also biometric parameters and the characterisation of the plants’ redox status and AOX machinery, allowing us to understand how these pathways were affected by stress factors and the genotype background. First, it was necessary to understand whether the mutant line would still present residual *AtExo70E2* expression. Indeed, the expression of the gene was greatly decreased when compared to the wt plants, but it was not a knockout, indicating that although the exocyst-mediated route would be impaired, it would not be fully abolished. The initial development of seedlings was then evaluated, and its effect on primary root length was assessed. Concerning the seedlings of the *exo70e2* line, several differences were observed regarding the stress treatments and the control. The aerial part of the seedlings in the C situation was smaller than in the wt plants. In general, all stress treatments affected the normal development of the seedlings, especially S1 and H2, where an extreme developmental delay was recorded. Regarding Zn-mediated stress, growth inhibition was observed, accompanied by the appearance of chlorotic spots. Interestingly, in the case of this mutant, the presence of H_2_O_2_ in the medium caused a delay in seedlings’ development, which was not observed for the wt plants. An overall reduction in the root length was found among treatments, apart from salinity and H1 treatment, where a significant increment was found. This observation has previously been described, and the data presented in the biometric parameters corroborate what is already described in the literature [25,26]. Altogether, data from the biometric assessment demonstrate that the EXPO is an important stress response mechanism [4,17], since there are notorious developmental delays in its absence. Symptoms associated with adverse environmental conditions, such as toxicity induced by ion excess, metals, and ROS, can occur at different levels, affecting the photosynthetic machinery and photosynthesis itself. For this reason, total chlorophylls and carotenoids were evaluated as photosynthetic markers. Starting with the wt seedlings, significant differences were found when seedlings were exposed to salinity (S1), drought (H2), and Zn treatments, although in terms of macroscopic manifestations, chlorosis was only found upon exposure to Zn. Metals may induce a reduction in the photosynthetic pigments, since they negatively affect the main source of energy production in plants, by restraining photosystem II activity and inhibiting the reduction in chloroplast enzymes as well as the photoreaction [27,28]. In addition, Na^+^ and Cl^-^ interfere with photosynthesis by affecting stomatal behaviour as well as through a combination of osmotic and ionic components, reducing the photosynthetic kinetics and photosynthetic capacity, respectively [29]. Overall, similar results were found for *exo70e2* plants, though specific differences could be noticed. In S1, seedlings did not show any reduction in total chlorophylls, yet they were affected by H_2_O_2_ exposure, contrary to the wt. It is known that the lower levels of pigments in response to oxidative stress may be related to ROS action in the chloroplasts, namely H_2_O_2_, that inhibit photosynthetic machinery and their counterparts [30]. 

### 3.2. Relative Expression of EXPO-Related Genes

An alternative autophagic pathway, mediated by the exocyst-positive organelle (EXPO), has been described as having a major role in plant tolerance to adverse conditions [4]. A previous study showed that the exocyst pathway was enhanced in relation to the autophagic route when seedlings were grown under abiotic stress conditions [12]. Thereby, in the present work, we assessed how genes associated with EXPO function and the autophagic pathway responded to the absence of one of the essential factors for the formation of the exocyst, the EXO70E2. The expression of *AtATG8*, a well-known marker for intracellular autophagy [31]; of *AtVTI12*, which encodes a protein responsible for the fusion of the vesicles of both EXPO and autophagic pathways with the vacuole; and of *AtSYP51*, a SNARE involved in the exocytosis process, were evaluated in this mutant background. To obtain an integrated view of the results obtained, two different analyses were made—(1) a comparison of the genes’ expression under abiotic stress with the control and in the mutant background, and (2) a comparison of the gene expression in the mutant with the same condition in wt plants. 

Starting with the analysis of the stress conditions in the mutant background, and given the results previously obtained in our laboratory [12], an upregulation of *AtVTI12* was expected. VTI12 is a SNARE protein involved in several cellular processes, namely in the docking and fusion of transport vesicles to the vacuole and to the plasma membrane [32,33], which also participates in the fusion of autophagic and EXPO vesicles to the tonoplast [16]. However, *AtVTI12* also did not change its expression in response to stress, indicating that when facing adverse environmental conditions, the lower expression of *EXO70e2* presents a small interference with the expression of *AtVTI12*. On the other hand, the comparison of the mutant plants with the wt ones showed that *AtVTI12* is upregulated in S and H1 stresses. This could be related to the role of VTI12 in the autophagy pathway for the degradation of cellular contents [34]. Another possibility, since S and H1 mimic osmotic stress conditions, relies on the role of VTI12 in maintaining cellular homeostasis, as it needs to be enhanced when the EXPO route is compromised. Moreover, it is also worth mentioning the augmented expression of *ATVTI12* in control conditions compared to wt plants (*p* value = 0.06). This observation indicates that EXO702E2, or the exocyst itself, may interfere with *VTI12* expression, being a negative regulator of its expression in normal physiological conditions. If that is the case, it would be worth exploring this pathway in more detail, in addition to the putative connection between the VTI12 and the EXPO vesicles. 

Autophagy is one of the main processes for maintaining cell homeostasis during abiotic stress responses [35,36,37,38], and it is closely related to exocyst activity [39]; thus, there is interest in evaluating the expression of *AtATG8* in the *exo70e2* mutant background. In our study, *AtATG8* did not alter its expression in *exo70e2* plants when compared to the control, except in the S and H1 and H2 situations, indicating that autophagic processes may have a more relevant role in osmotic-related stress, where the sequestering and storage of ions and other molecules is very important for maintaining cell homeostasis. In fact, the association of autophagy with drought and salt stress has already been documented [38], with authors concluding that autophagy can be regulated by distinct signalling pathways in response to different environmental conditions. These data pair with the augmented gene expression of *AtATG8* in H2 compared to in wt plants. In contrast, comparing the expression in the mutant background with wt values, the fact that *AtATG8* is downregulated in Ox and Zn conditions can be related to the fact that selective autophagy processes, such as pexophagy (a type of macroautophagy that selectively degrades peroxisomes), are being activated instead of the typical autophagic process [40]. The levels of the stress markers and the inhibition of the enzymatic activity on *exo70e2* plants corroborate it, since peroxisomes contain multiple AOX enzymes, especially CAT [41]. Nevertheless, in vivo studies regarding these two pathways are needed to confirm this hypothesis. Altogether, these results indicate that autophagic processes are not completely independent from exocysts, as *AtATG8* expression varies in the mutant background. It is also clear that autophagic processes do not increase in the absence (or lower quantities) of EXPO vesicles. In fact, the downregulation of *ATG8* in the control situation compared to wt seems to indicate otherwise and corroborates the hypothesis that these two pathways may be interconnected. 

SYP51 is usually involved in post-Golgi membrane trafficking to the tonoplast [42,43], which is also associated with stress events, given its interaction with aquaporins [42]. Considering the stress situations under study in the mutant background, the expression of *AtSYP51* presents a significant upregulation in H1, H2, and Zn conditions. This may be linked with the role of the encoded protein in exocytosis processes [43] or with its interactions with other proteins, such as NIP1.1, an ER aquaporin, at the tonoplast [42]. Aquaporins are membrane channels that facilitate water transport in several cellular compartments [44], and they could explain the upregulation of *AtSYP51* in drought stress conditions. Similarly, comparing the expression of *AtSYP51* in the mutant with the wt plants, only H1 induced a significant overexpression. This upregulation occurred in an osmotic stress condition, and the role of aquaporins in maintaining the cellular osmotic state is quite well established [44].

Combining these results with the morphological traits of the *exo70e2*, namely the delayed development in salinity and drought situations, a clearer image of the importance of the EXPO-mediated transport for stress response was obtained. However, other genes participating in this process should be tested further to evaluate how the exocyst formation may or may not occur, or whether other non-conventional pathways are preferred.

### 3.3. Characterisation of the AOX Response in the exo70e2 Background

ROS generation is enhanced upon adverse environmental conditions, leading to an overaccumulation of these compounds [5,6]. Although they can act as important intracellular signalling agents at low doses, ROSs become phytotoxic at high concentrations, affecting the overall plant cell homeostasis. Therefore, to regulate the cellular concentrations of ROS, the AOX system is activated to re-establish redox homeostasis [7,8]. In this study, several biochemical assays were conducted to evaluate some oxidative stress indicators (H_2_O_2_ and LP), along with the AOX system’s response, to better understand how the protein sorting machinery directly affects the oxidative stress response.

H_2_O_2_ is regarded as one of the primary ROSs, and is also considered a key signal in different physiological mechanisms [5]. In general, different responses were found between *exo70e2* and wt plants in different stress conditions, revealing that H_2_O_2_ levels were modulated by both factors (genotype and abiotic stress). First, no major changes were found between the genotypes under control conditions, revealing that ROS content did not seem to be altered, at least under homeostasis-promoting conditions. Moreover, in the wt seedlings, no major changes in ROS content were found in response to stress, except in the drought treatment, where its levels decreased, and in the Ox stress, where, expectedly, its content rose significantly. This result suggests an uptake of H_2_O_2_ from the nutritive medium and/or an intracellular overproduction in response to the external stimuli. As a result of ROS overproduction, LP can occur, damaging the biological membranes. Indeed, this process may aggravate the cellular redox imbalance, since radicals derived from the LP can react with proteins and DNA [8]. Following the pattern registered for H_2_O_2_, MDA levels in wt seedlings were only enhanced in response to the Ox treatment, revealing the occurrence of great redox disorders. On the contrary, *exo70e2* plantlets showed no increase in H_2_O_2_ content and LP degree; actually, their content was even reduced in almost all situations. Minimal levels were observed in the *exo70e2* mutant line when exposed to Ox and Zn, which was unexpected, since peroxidation of lipids is a common metabolic event resulting from plants’ exposure to metal excess [45]. Here, a new hypothesis can be raised—the AOX system is more efficiently activated in the mutant than in the wt, lowering the overaccumulation of ROS and the lipid peroxidation degree. Indeed, this seems to be the case, as the mutant exhibited a general decrease in H_2_O_2_ and LP content in almost all stress conditions. Following this line of thought, several other authors focusing on abiotic stress (water stress, metal toxicity and oxidative damage) have reported that decreases in ROS content are often related to improved efficiency of the AOX system, allowing better acclimation of the plants to stress [46,47,48].

To explore the involvement of the non-enzymatic AOX system, the accumulation of Pro and GSH was quantified. Proline accumulation is a common response of plants to a large range of stress factors, due to its multiple biological functions. The proteinogenic amino acid can act as an osmoprotectant, conferring tolerance to water-related stresses, but is also involved in the stabilisation of biological membranes, scavenging of ROSs, and metal sequestration [5]. Thus, changes in Pro levels are often considered early warning signals of oxidative stress, and provide an overall assessment of the cellular responses to different stressors [5,27,49]. In the wt plants, substantial rises in Pro were expected, especially under S and H2 treatments, given its well-recognised functions as an osmolyte and compatible solute [50]. Still, even in other stress conditions, such Ox and Zn, Pro accumulation also took place, indicating that induction of Pro production is a typical response for Arabidopsis plants under stress [50,51,52]. Equivalent findings were also reported for tomato plants exposed to nickel and drought stress [34]. However, the mutant behaves somewhat differently: *exo70e2* only increased the levels of Pro in S and H2 treatments. As such, it is possible that, at least in this mutant, Pro functions mainly as an osmolyte rather than an AOX, and that other defence mechanisms are being activated to balance ROS overproduction. GSH is an important cellular redox buffer since it plays a role in different components of the AOX system, such as the AsA-GSH cycle and Glutathione reductase activity, and also participates in processes linked to protein synthesis, enzymatic regulation, and expression of stress-responsive genes [5]. Curiously, the levels of GSH in control plants were very distinctive between genotypes, since the GSH content in wt was four-fold higher than in the mutant. Moreover, both genotypes showed an increase in GSH levels in Zn, a result which was expected, since this molecule is also a metal chelator [5]. Additionally, the *exo70e2* line also showed large GSH increases under H2 and Ox stresses, situations where Pro was not enhanced, which can be correlated with the fact that this molecule has other functions besides being a metal chelator, such as being the subtract for the glutathione S-transferases (GST; EC 2.5.1.18) [53,54].

To gain better insight into the full picture of the ROS detoxification, enzymatic players of the AOX system were also evaluated. For this purpose, CAT and APX, both related to H_2_O_2_ detoxification, were analysed. In *exo70e2* seedlings, there was a clear tendency to decrease the activity of APX and CAT enzymes, which became significant in S1-treated seedlings. In this case, it is also important to hypothesise that the AOX enzymes in this mutant are not particularly relevant to the seedling’s response, since this mutant appears to be investing in the non-enzymatic players, such as Pro and GSH. Moreover, the inhibition of CAT and APX activities in the *exo70e2* mutant can also be linked to H_2_O_2_-mediated impairment of enzyme activity, as these seedlings show developmental delays in situations where the amount of H_2_O_2_ does not increase, so there is no need for the increase in these enzymes’ activity. Either way, especially in terms of physiological performance and redox status, mutant plants seem to be able to cope more effectively with stressful conditions than wt plants. 

Overall, with all the data presented so far, it is possible to observe that the mutant line activated distinct physiological responses to deal with the abiotic stresses from the wt. Moreover, *exo70e2* seedlings demonstrated a preference for the non-enzymatic AOX system, as the activation of these important players was coupled with the reduced ROS levels and LP degree, despite no differences being observed in the enzyme activity. Still, in terms of growth performance, this mutant exhibited significant differences from the wt, showing reduced growth and development, and revealing that the high energy investment in the defence mechanism may have compromised the allocation of resources to growth.

## 4. Materials and Methods

### 4.1. Germination and Stress Experiments

*Arabidopsis thaliana (col 0)* T-DNA line for *exo70e2* was ordered from NASC (Nottingham Arabidopsis Stock Centre, Loughborough, United Kingdom—SAIL_637_D01). Seeds were germinated in half-strength Murashige and Skoog medium (MS) (Duchefa, Haarlem, The Netherlands), supplemented with 1.5% (*w/v*) sucrose, and solidified with 0.7% (*w/v*) bacteriological agar. In order to test the effects of different abiotic stress factors, wild type (wt) and T-DNA lines were grown under in vitro conditions in the above cited media and supplemented with 50 mM NaCl (S, saline stress), 50 (H1) and 100 (H2) mM mannitol (drought stress), 0.5 mM hydrogen peroxide (H_2_O_2_) (Ox, oxidative stress), and 150 µM zinc sulphate (ZnSO_4_.5H_2_O) (Zn, metal toxicity) (Table 1). The selected concentrations applied were based on recent work [12]. Seeds were maintained for 48 h at 4 °C, in the dark to promote stratification, and were then transferred to a growth chamber under a photoperiod of 16 h light/8 h dark, an average humidity of 50–60%, and a temperature of 21 °C, with a light quantity of 110 μmol m^−2^ s^−1^, for 10–12 days. Four biological replicates were prepared for each stress situation. After this period, the seedlings were collected, the root length was measured, and they were frozen in liquid nitrogen before the subsequent analysis was performed.

### 4.2. Biochemical Assays

In order to assess the physiological and biochemical responses of the selected mutants, several biomarkers were characterised.

#### 4.2.1. Photosynthetic Pigments Quantification

The quantification of total chlorophyll (chl) and carotenoids was performed according to Lichtenthaler (1987). Aliquots of 80 mg of tissue were homogenised with 80% (*v/v*) acetone. The homogenates were then centrifuged at 1400× *g* for 10 min, and the SN was collected and maintained in the dark. The absorbance was read at 470, 647, and 663 nm. The levels of chl a, chl b, and carotenoids were calculated from formulas derived by Lichtenthaler (1987), and the final results were presented in mg g^−1^ fresh weight (fw).

#### 4.2.2. Determination of Lipid Peroxidation (LP)

Lipid peroxidation (LP) was evaluated in accordance with Heath and Packer (1968) through the quantification of malondialdehyde (MDA) levels, as it is an end-product formed from polyunsaturated fatty acids peroxidation. Aliquots of 100 mg were homogenised in 0.1% (*w/v*) trichloroacetic acid (TCA) with the use of Bead Ruptor 12 (Omni International, Kennesaw, GA, United States), a bead-miller homogeniser. To prevent overheating of the samples, three homogenisation cycles (5 m s^−1^; 20 s) were performed with a one-minute period on ice between each. Then, samples were centrifuged for 15 min at 10,000× *g*. After recovering the SN, 1 mL of 0.5% (*w/v*) thiobarbituric acid (TBA) in 20% (*w/v*) TCA was added to 250 µL of SN. This mix was incubated for 30 min at 95 °C, followed by incubation on ice for 15 min. An 8-minute centrifugation at 10,000× *g* was performed, and the absorbance of the samples was measured at 532 and 600 nm. The latter result was subtracted from the first to avoid the effects of non-specific turbidity, by utilising the extinction coefficient of 155 mM^−1^ cm^−1^. MDA content was expressed as nmol g^−1^ fw.

#### 4.2.3. Quantification of H_2_O_2_

Hydrogen peroxide (H_2_O_2_) levels were quantified according to Alexieva and co-workers (2001), with few modifications for adapting to a microplate. Briefly, frozen aliquots of 100 mg of seedling tissue were homogenised in 1 mL of 0.1% TCA using a bead miller homogeniser (Bead Ruptor 12, OMNI International, Kennesaw, GA, United States). To prevent overheating of the samples, three homogenisation cycles (5 m s^−1^; 20 s) were performed with a one-minute period on ice between each. The homogenates were centrifuged at 12,000× *g* for 15 min at 4 °C. The SN was used to perform the rest of the protocol, were 50 µL were removed and transferred to each well of the microplate. Then, 50 µL of PK buffer (100 mM, pH 7.0) and 200 µL of 1 M potassium iodide (KI) were added. The mix was left in obscurity at RT for 1 h, and the absorbance was read at 390 nm. The final results were expressed in nmol g^−1^ fw, using 0.28 µM cm^−1^ as the extinction coefficient. 

#### 4.2.4. Determination of Proline (Pro) Levels

The quantification of proline (Pro) levels was performed as described by Bates and co-workers (1973). Frozen seedling samples were homogenised in 3% (*w/v*) sulfosalicylic acid in the Bead Ruptor 12 (Omni International, Kennesaw, GA, United States). To prevent overheating of the samples, three homogenisation cycles (5 m s^−1^; 20 s) were performed with a one-minute period on ice between each. Then, the homogenates were centrifuged at 16,000× *g* for 10 min, and the SN recovered. Afterwards, 200 µL of each sample was mixed with 200 µL of glacial acetic acid and 200 µL of acid ninhydrin. This was followed by incubation at 95 °C for 1 h. After the samples were cooled out on ice, 1 mL of toluene was added, and the mixture was vortexed for 15 s to separate a red upper phase (organic) from a whitish lower phase. The upper phase is utilised to perform the absorbance reads at 520 nm. Pro concentration and absorbance were linked via a standard curve designed with known increasing concentrations of this metabolite, and results were expressed in mg g^−1^ fw.

#### 4.2.5. Determination of Reduced Glutathione (GSH)

To quantify glutathione (GSH) levels, a protocol adapted from Soares and colleagues (2019) was used. Briefly, 1 mL of 3% (*w/v*) sulfosalicylic acid was used to homogenise 100 mg of frozen tissue in the Bead Ruptor 12 (Omni, International, Kennesaw, GA, United States) homogeniser, followed by centrifugation at 16,000× *g* at 4 °C for 10 min. To prevent overheating of the samples, three homogenisation cycles (5 m s^−1^; 20 s) were performed with a one-minute period on ice between each. The SN was collected, then 50 µL of it was added to 200 µL ddH_2_O and 750 µL of a reaction mixture containing 100 mM PK (pH 7.0), 10 mM EDTA, and 1.5 mg mL^−1^ 5,5-dithio-bis-(2-nitrobenzoic acid) (DTNB). The mixture was vortexed and incubated in the dark for 10 min. The absorbance was read at 412 nm, and the levels of GSH were quantified according to a standard curve prepared with known concentrations of this AOX. The results were expressed in µmol g^−1^ fw. 

#### 4.2.6. Determination of Catalase (CAT) and Ascorbate Peroxidase (APX) Activity

##### Extraction and Quantification of Soluble Proteins

To quantify total soluble proteins, aliquots of 100 mg of frozen tissue were homogenised, with the aid of Bead Ruptor 12 (Omni International, Kennesaw, GA, United States), in 2 mL of potassium phosphate (PK) buffer (100 mM, pH 7.3), supplemented with 1 mM EDTA, 1 mM phenylmethylsulphonyl fluoride (PMSF), 5 mM L-ascorbic acid, 8% (*v/v*) glycerol, and 1% (*w/v*) polyvinylpolypyrrolidone (PVPP). To prevent overheating of the samples, three homogenisation cycles (5 m s^−1^; 20 s) were performed with a one-minute period on ice between each one. The homogenates were centrifuged at 4 °C for 25 min at 16,000× *g,* then the SN was collected and used for both protein quantification and enzyme activity. Total protein was quantified based on the protocol described by Bradford (1976) [55]. For that, a 1:100 dilution of each SN was obtained. Then, 75 µL of the diluted sample was mixed with 750 µL of Bradford Solution (PanReac AppliChem, Barcelona, Spain), followed by a 15 min incubation period in the dark. Finally, the absorbance was read at 595 nm.

##### Determination of CAT and APX Activity

Regarding APX, its activity was evaluated based on the protocol of Nakano and Asada (1981), adapted for UV-microplates by Murshed and colleagues (2008). Briefly, 20 µL of the protein extract was mixed with 170 µL PK (50 mM, pH 7), containing 0.6 mM AsA. Immediately before the measurement, 10 µL H_2_O_2_ (254 mM) were added to each well of the UV-microplate. APX-mediated AsA oxidation was followed at 290 nm every 5 s, with a 2 min interval. Utilising AsA molar extinction coefficient (0.49 mM cm^−1^), final activity values were expressed as µmol AsA min^−1^ mg protein^−1^.

Concerning CAT, the original procedure of Aebi (1984) was used and adapted for microplates. The temporal catalase-mediated degradation of H_2_O_2_ was measured spectrophotometrically. In a UV-microplate, 20 µL of SN were mixed with 160 µL of PK (50 mM, pH 7). Prior the absorbance reading, 20 µL of 100 mM H_2_O_2_ were added to each well. The CAT activity was followed in intervals of 5 s for 2 min, at 240 nm. Final activity values were determined with H_2_O_2_ molar extinction coefficient (39.4 mM cm^−1^) and expressed in µmol H_2_O_2_ min^−1^ mg protein^−1^. 

All of the procedures were performed in the microplate reader MultiskanGo (Thermo Scientific, Waltham, MA, USA), and the software used to analyse the data was SkanIt 6.1 (Thermo Scientific, Waltham, MA, USA).

### 4.3. Gene Expression Analysis by qPCR

To evaluate the expression of genes that encode for proteins involved in the related trafficking pathways as the gene targeted in the T-DNA line, RNA was extracted, cDNA produced and then qPCR assays were performed.

#### 4.3.1. RNA Extraction

To extract RNA from the *Arabidopsis* T-DNA lines, the Total RNA Isolation kit (NZYTech, Lisboa, Portugal) was used. The manufacturer protocol was used; however, the mass of plant tissue used was 150 mg and had previously been frozen in liquid nitrogen, and the elution volume was down to 20 µL. After the extraction, the RNA was quantified in a DS-11 microvolume Spectrophotometer (Denovix, Wilmington, NC, USA) and stored at −80 °C. 

#### 4.3.2. cDNA Production

To produce cDNA from the isolated RNA, the NZY First-Strand cDNA Synthesis kit (NZYTech, Lisboa, Portugal) was used according to the manufacturer’s protocol. The cDNA solution was stored at −20 °C.

#### 4.3.3. Real-Time Assays

All of the primers were already available in the laboratory (Table 2) and were already optimised, according to Neves et al. (2021), as well other reaction optimisations such as dilution tests, efficiency studies, and validation of the reference genes (*GAPDH* and *SAND*). The plate design was performed with the software Bio-Rad CFX Maestro 1.0 (BioRad, Hercules, CA, USA).

The qPCR reaction included 8 µL of a master mix composed of 315 µL of NZYSupreme qPCR Green Master Mix (2×) (NZYTech, Lisboa, Portugal), 400 nM of each primer (forward and reverse), 163.8 µL of water, and 2 µL of cDNA previously diluted 1:8, performing a reaction volume of 10 µL. The protocol used the following amplification conditions: initial denaturation (95 °C for 3 min); 40 cycles of amplification and quantification (95 °C for 10 s, 56 °C for 10 s and 72 °C for 30 s, with a single fluorescent measurement) and melting curve generation (65 °C to 95 °C, with one fluorescence read every 0.5 °C). The equipment used was CFX96 Real-Time System (BioRad, Hercules, CA, USA). Using Bio-Rad CFX Maestro (version 1.0) software, the cycle threshold (Ct) and expression tests were calculated by comparing them with control conditions. 

### 4.4. Statistical Analysis

All biometrical and biochemical procedures were performed in at least three independent biological replicates (n ≥ 3). Prior to any statistical treatment, variance homogeneity was checked by the Brown–Forsythe test. Analysis with only one variable was examined through a one-way ANOVA, and qPCR assays and biochemical assays were only performed regarding stress situations, while the tests comparing two variables (genotype and stress) used a two-way ANOVA. All of the multiple comparisons were treated with a Dunnett’s multiple comparison tests. All statistical analyses were performed in Prism 8 (GrahPad^®^, San Diego, CA, USA).

## 5. Conclusions

Many studies devoted to understanding how protein sorting affects the stress response or participates in it do not draw a clear picture of how this process impacts plant behaviour, as the responses of other metabolic pathways are important for plant adaptation. By combining functional genetics, biochemistry, and gene expression data, this work is pioneering, as it presents the first overview on how sorting machinery directly affects the plant at the physiological and molecular levels. Although working on such a topic is quite important, it is also challenging, since no major conclusions can be drawn at the moment without further studies.

The main results of this study rely on the fact that *exo70e2* preferred the non-enzymatic AOX component. The correlation between the mutant and the response from the AOX system is not immediate, as little is known regarding the trafficking of these molecules. Nevertheless, the results obtained herein allow us to speculate that the EXPO may have a role in the transport of AOX enzymes from the cytoplasm towards other locations in the cell. In its absence, it is fair to assume an impairment of these transports in the mutant. In fact, more than two decades ago, Mullen and co-workers (1999) [56] demonstrated that plant peroxisomal APX is sorted to peroxisomes via a side pathway, including a preperoxisomal compartment with traits of a distinctive ER subdomain, potentially a peroxisomal ER subdomain. The exocyst was only discovered many years later, so it would be beneficial to revisit the biogenesis and trafficking of these enzymes and to cross it with the vast knowledge and tools now available for studying endomembrane trafficking. Additionally, it seems evident that a knockdown of *EXO70e2* affects genes associated with stress responses, causing its upregulation in situations leading to osmotic stress (salinity and drought). As such, it is logical to assume that EXPO may have a role in maintaining cellular homeostasis, likely by sequestering ions and molecules from the cytoplasm. When the cell has fewer available EXPO vesicles, it activates other mechanisms to cope with the imbalance in cellular homeostasis.

As a whole, and additionally to the data already produced with this project, this study demonstrates promising results for future research and opens doors for bringing these two areas together in the study on plants’ adaptation/tolerance to stress.

## Figures and Tables

**Table 1:** Different conditions and stress inducers used to simulate abiotic stress.

Stress Type	Nomenclature	Stress Inducer	Concentration
Control	C	-	-
Saline	S	Sodium Chloride	50 mM
Hydric	H1	Mannitol	50 mM
	H2	Mannitol	100 Mm
Oxidative	Ox	Hydrogen Peroxide	0.5 mM
Zinc	Zn	Zinc Sulphate	150 µM

**Table 2:** List of genes and corresponding primer pairs used in the quantitative RT-PCR assay.

Genes	Primer Forward	Primer Reverse
*SAND* ^1^	AACTCTATGCAGCATTTGATCCACT	TGATTGCATATCTTTATCGCCATC
*GAPDH* ^1^	TTGGTGACAACAGGTCCAAGCA	AAACTTGTCGCTCAATGCAATC
*EXO70*	TCCCCGATGAAACAGGCTCGTC	GCCTCCATGAAAGGGGCGTGT
*ATG8*	TTGCTTGCTTGAAATTCGCA	TTCACTCATCCTTGCCTCGA
*VTI12*	GCAATGTCCGTGGAGAGGCTTGA	TGCGCATGAAGGAGGGTTTGG
*SYP51*	TGGCGTCTTCATCGGATTCATGG	AGCTGAAGCACGACGCTGAGCA

^1^ from (Czechowsaki et al., 2005).

## Data Availability

Not applicable.
